# Incidence and risk factors of graft-versus-host disease after liver transplantation: A national study 2010–2020

**DOI:** 10.1097/HC9.0000000000000271

**Published:** 2023-09-27

**Authors:** Yuting Huang, Yichen Wang, R. Christopher Chase, Liu Yang

**Affiliations:** 1 Department of Gastroenterology & Hepatology, Mayo Clinic, Jacksonville, Florida, USA; 2 Mercy Internal Medicine Service, Trinity Health of New England, Springfield, Massachusetts, USA; 3 Department of Medicine, Mayo Clinic, Jacksonville, Florida, USA; 4 Division of Hepatology and Liver Transplantation, Mayo Clinic, Jacksonville, Florida, USA

## Abstract

**Background:**

Graft-versus-host disease (GVHD) is a common complication of hematopoietic cell transplantation, and its incidence is low in liver transplantation (LT). Estimating the incidence of GVHD after LT is challenging due to the paucity of available data from the United Network for Organ Sharing. This is the first national analysis of the incidence and risk factors of GVHD after LT.

**Methods:**

This retrospective cohort study used the National Readmission Database to calculate the incidence rate of GVHD within 1 year of LT using survival analysis. The predictors of GVHD were identified using univariate and multivariate Cox regression analyses.

**Results:**

From 2010 to 2020, of 88,433 LTs, 383 cases of GVHD occurred within 1 year after LT, resulting in an incidence rate of 1.0% (95% CI: 0.8%–1.3%). We observed no statistically significant change in the incidence of GVHD after LT from 2010 to 2020 (beta-coefficient, −0.07%; 95% CI: −0.17% to 0.04%, *p* = 0.188). Interestingly, alcohol-associated liver disease was associated with a lower risk of GVHD (adjusted HR, 0.57; 95% CI: 0.36–0.91, *p* = 0.018), whereas a higher risk was found to be related to a secondary diagnosis of COVID-19 on index admission.

**Conclusion:**

Our study found that the incidence rate of GVHD within 1 year of LT in the United States was 1.0% and remained stable from 2010 to 2020. The predictors associated with GVHD include alcohol-associated liver disease and COVID-19. Our study provides valuable insights into the incidence, risk factors, and outcomes of GVHD after LT.

## INTRODUCTION

**FIGURE 1:**
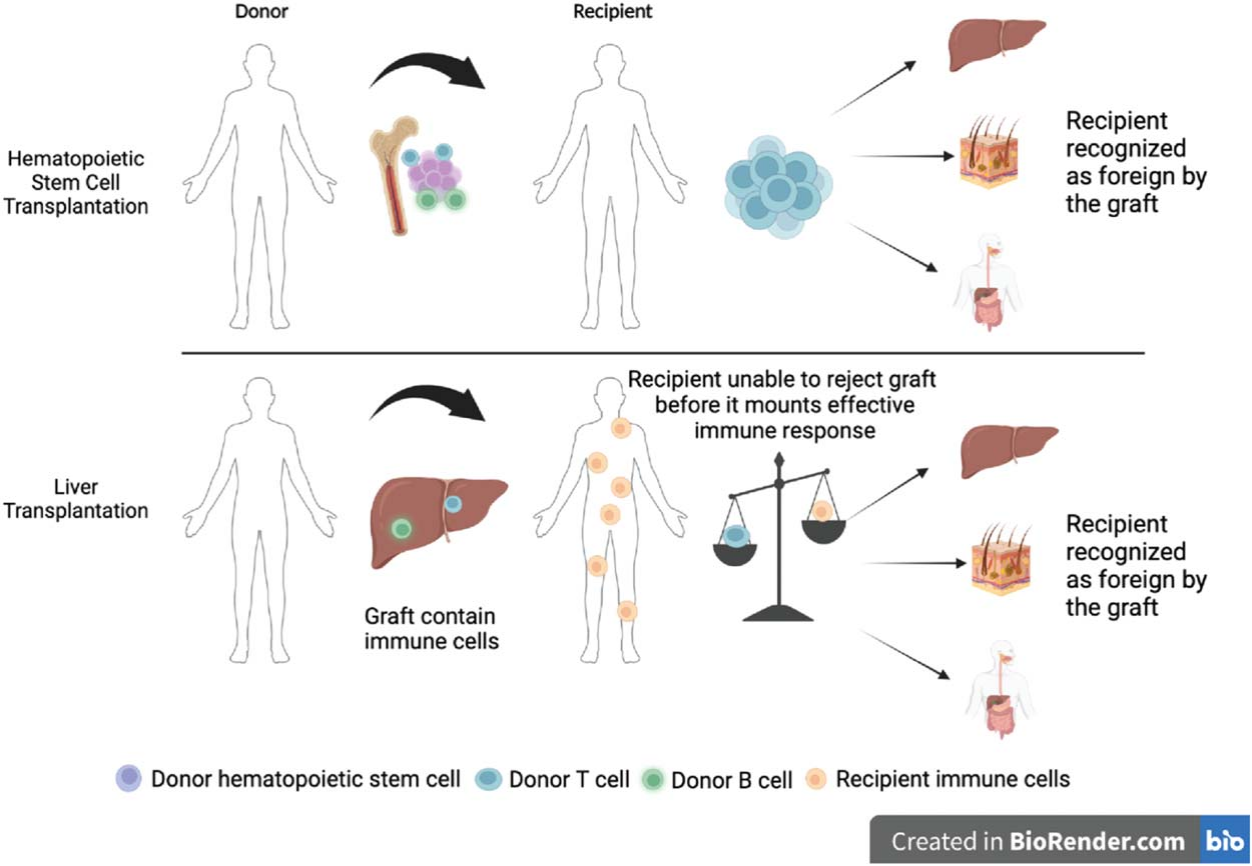
Comparative illustration of GVHD mechanisms in HSCT versus LT. The top panel outlines the typical process in HSCT whereby donor T cells mount an immune response against host antigens, triggering a cytokine release with resultant local and systemic tissue damage. The bottom panel contrasts this with a hypothetical mechanism in GVHD after LT. Here, donor lymphocytes, accompanied by the transplanted liver as passenger cells, are assumed capable of initiating an immune response against the host tissues. Abbreviations: GVHD, graft-versus-host disease; HSCT, hematopoietic stem cell transplantation; LT, liver transplantation. The figure is created with BioRender.com.

Graft-versus-host disease (GVHD) is a significant complication that occurs frequently after hematopoietic cell transplantation but is less common following liver transplantation (LT) and other solid organ transplantations (Figure 1). Estimating the incidence of GVHD after LT in the United States is challenging because of the absence of GVHD post-transplant data collection and reporting by the United Network for Organ Sharing (UNOS).^1^ Current GVHD incidence estimates are derived from single institutional studies and range from 0.1% to 2%; however, the national incidence of GVHD after LT is unknown.^1–7^ Given the severity of GVHD and the limited understanding of its incidence after LT in the United States, a more comprehensive assessment of its national incidence and associated risk factors is necessary.

GVHD occurs when transplanted immune cells from a nonidentical donor (the graft) recognize the recipient (host) as foreign, thereby initiating an immune reaction that can cause severe disease. Livers contain high numbers of donor lymphocytes, making GVHD after LT relatively common compared with other solid organ transplants.^1,8^ GVHD following LT can present with a range of symptoms including skin rash, gastrointestinal distress, liver dysfunction, and bone marrow suppression.^9–13^ The mortality rate of GVHD after LT is reportedly to be as high as 85%.^3,5^ With new treatment methods being developed, the mortality rate with treatment of GVHD after LT with steroids alone remains 84%, 75%–100% with regimens using dose increases of calcineurin inhibitors, and 55% with IL-2 antagonists.^3,5^ With over 8000 LTs performed in the United States each year and a 1-year survival rate of over 90%,^14^ a more thorough understanding of this serious complication is critical for optimizing the longevity of LT recipients.

In this study, we aimed to explore the incidence of GVHD after LT and the associated risk factors using the National Readmission Database (NRD). NRD is currently the most extensive database for readmission tracking after LT index admission. The NRD records 17 million discharge events annually and offers a unique opportunity to examine rare events, such as GVHD after LT, which could not be accomplished at individual institutions, as seen in previous studies. Furthermore, by adopting the survey methods provided by the Agency for Healthcare Research and Quality, the NRD provides nationally representative data, enhancing the generalizability of our findings to patients throughout the United States.

## METHODS

### Study design and database description

This was a retrospective cohort study of patients hospitalized for LT at acute-care hospitals across the United States from 2010 to 2020. The NRD is the largest publicly available all-payer readmission database in the United States and was used to extract readmission data associated with GVHD following LT. The NRD was created by the Agency for Healthcare Research and Quality as a part of the Healthcare Cost and Utilization Project, and its methods have been described elsewhere.^15^ With data from over 17 million discharges annually, the NRD is a powerful tool to leverage when examining uncommon conditions such as GVHD after LT. The use of the Agency for Healthcare Research and Quality survey methods in this study allows for nationally representative results. The dataset for each year includes data from January through December. The first reported case of COVID-19 in our data was in April 2020, which is consistent with the timeline of the implementation of the International Classification of Diseases, 10th Revision (ICD-10) code of COVID-19. The use of limited datasets in this study did not require an institutional review board review under the Health Insurance Portability and Accountability Act.^16^

### Identifying variables

The International Classification of Diseases 9th Revision (ICD-9)/ICD-10 codes were used to identify various disorders and symptoms (Supplemental Table S1, http://links.lww.com/HC9/A536). LT and GVHD information was extracted using the ICD-10 codes “0FY00Z0” and “D89.81,” respectively. We expanded our analysis to investigate liver disease as a GVHD risk factor after hematopoietic stem cell transplantation (HSCT), using sufficient sample data from 2016 to 2020.

To characterize the patient population, we extracted the following variables from the NRD: age, sex, race, median household income, and primary expected payer (ie, insurance). The comorbidity burden was quantified using the Elixhauser index, a scoring system that includes a list of 30 comorbidities, which is widely used in health care research to control for the influence of comorbidities on outcomes and provides a standardized and quantitative measure of comorbidity burden.^17^ ICD-10 codes were used to identify liver diseases, including viral hepatitis, alcohol-associated liver disease (AALD), autoimmune hepatitis, and hepatocellular carcinoma (HCC). In-hospital all-cause mortality rates were determined using the discharge status in the NRD because the NRD does not specify the cause of death.

### Statistical analysis

Statistical analyses were conducted using Stata, version 17.0 (StataCorp). Continuous variables were analyzed using the Student’s *t* test if they were normally distributed and the Wilcoxon rank-sum test if they were not. Proportions were compared using the chi-square test. Survival analysis was conducted to determine the incidence rates. The incidence rate was defined as the number of new GVHD cases divided by the total person-time at risk (LT recipients) during the follow-up. The incidence proportion was the proportion of LT recipients who developed a new case of GVHD during follow-up. The follow-up period ended either on admission with a diagnosis of GVHD or death, or at the conclusion of each calendar year. All *p* values were 2-sided, with a significance level of 0.05.

## RESULTS

### Incidence and mortality of GVHD

**FIGURE 2:**
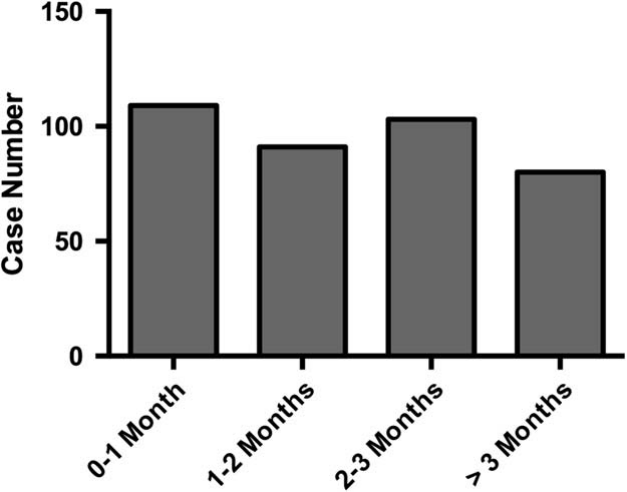
Incidence of GVHD after LT by time interval. The graph displays the number of incident cases of GVHD after liver transplantation, categorized by the time interval from the transplant to the onset of GVHD. The time intervals are divided into 4 categories: 0–1, 1–2, 2–3, and more than 3 months. The *y*-axis represents the number of cases, while the *x*-axis shows the time intervals. The data are presented as a bar graph, with each bar corresponding to a different time interval. Abbreviations: GVHD, graft-versus-host disease; LT, liver transplantation.

**FIGURE 3:**
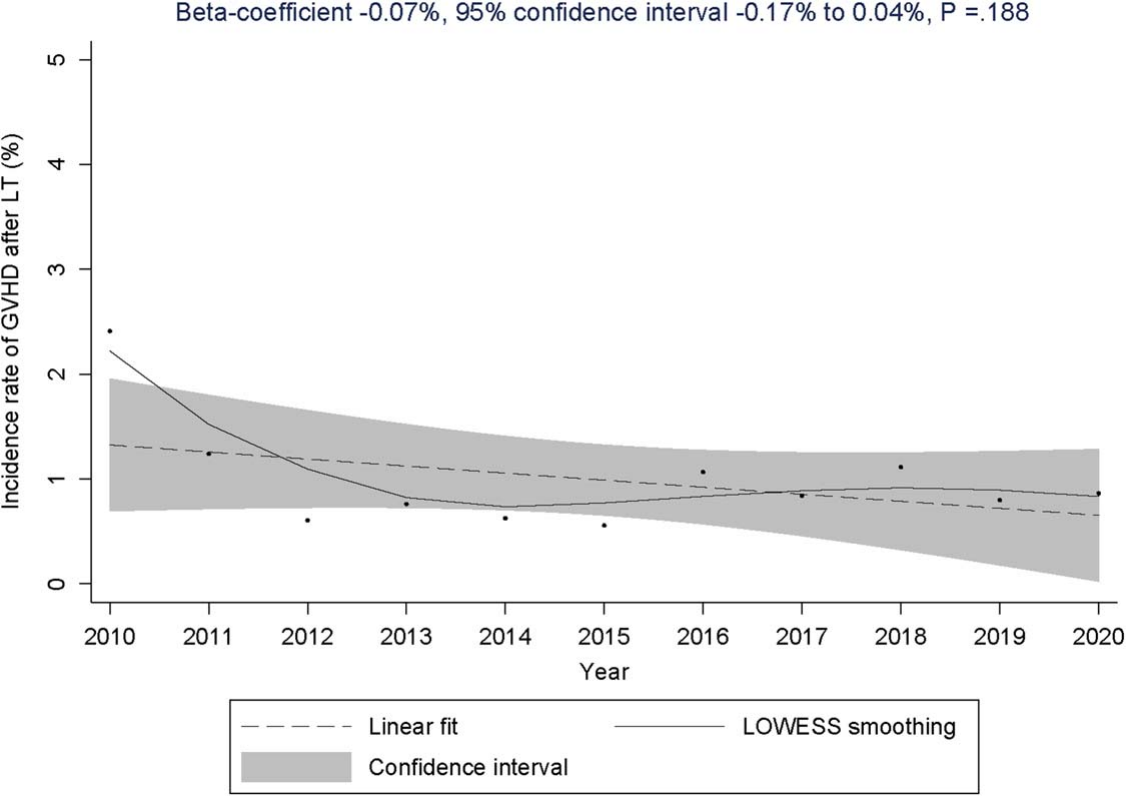
Trend of the incidence rate of GVHD following LT, 2010–2020. The result of the linear regression is displayed at the top of the graph, and the solid curve represents the LOWESS regression, which was used to show the smoothed relationship between years and incidence rates. Abbreviations: GVHD, graft-versus-host disease; LOWESS, locally weighted scatterplot smoothing; LT, liver transplantation.

A total of 88,433 LTs were performed from 2010 to 2020, of which 383 developed GVHD within a year. This revealed a GVHD incidence proportion of 0.4% at an average of 6.6 months of follow-up. The mean time from LT to GVHD development was 79.4 days, and the median was 50 days (interquartile range: 27–100 d). The incidence rate of GVHD after LT was 1.0% (95% CI: 0.8%–1.3%; Figure 2). From 2010 to 2020, the incidence rate of GVHD after LT varied from 0.6% to 2.4% (Table 1). Although there were year-to-year variations (eg, lower HR in the year 2020 compared with 2010 with HR 0.36, 95% CI: 0.14–0.90, *p* = 0.029; Supplemental Table S2, http://links.lww.com/HC9/A536), there was no statistically significant change in the trend from 2010 to 2020 (beta-coefficient: 0.07%, 95% CI: −0.17% to 0.04%, *p* = 0.188; Figure 3). The in-hospital and calendar-year all-cause mortality rates of GVHD hospitalization were 20.8% and 41.7%, respectively. In addition, 19.1% of patients who did not pass away on the index GVHD hospitalization received palliative care consultation within that calendar year.

**TABLE 1:** Incidence rate of GVHD following LT, 2010–2020

Year	LT	GVHD	Incidence rate (%)	95% CI
2010	3392	82	2.4	1.0%–7.6%
2011	3381	42	1.2	0.7%–2.6%
2012	3233	20	0.6	0.3%–1.3%
2013	3112	24	0.8	0.4%–1.5%
2014	3161	20	0.6	0.3%–1.3%
2015	3535	20	0.6	0.3%–1.3%
2016	3540	38	1.1	0.6%–2.0%
2017	3597	30	0.8	0.5%–1.6%
2018	3760	42	1.1	0.7%–2.0%
2019	3985	32	0.8	0.5%–1.4%
2020	3979	34	0.9	0.5%–1.5%
Total	38675	383	1.0	0.8%–1.3%

Abbreviations: GVHD, graft-versus-host disease; LT, liver transplantation.

Further analysis revealed that, among the first episodes of GVHD occurring after LT, 37.0% were coded as acute GVHD, 6.6% as chronic GVHD, and 1.2% as acute or chronic GVHD, and the remaining 55.2% were unspecified. This distribution suggests that most of the instances of GVHD in our dataset were either acute or unspecified. Furthermore, the bulk of these cases emerged within 3 months following LT (Figure 2), which is in line with the traditional definition of “acute GVHD.”

### Patient and hospital characteristics of LT hospitalizations

Table 2 presents patient characteristics of index hospitalizations for LT with or without subsequent GVHD. The sample consisted of 88,050 patients without GVHD and 383 patients with GVHD. The age of patients in both groups was similar, with a mean of 57 and 58 years, respectively (*p* = 0.338). The proportion of female gender was higher in the GVHD group (43.7%) compared with the non-GVHD group (35.4%), but this difference was not statistically significant (*p* = 0.282). The median household income in the patient’s zip code and the Elixhauser comorbidity index were also similar between the 2 groups. There were statistically significant differences in the proportion of patients with AALD (19.0% in the GVHD group vs. 29.0% in the non-GVHD group, *p* = 0.023) and in the proportion of patients with a secondary diagnosis of COVID-19 (9.7% in the GVHD group vs. 0.6% in the non-GVHD group, *p* < 0.001).

**TABLE 2:** Patient characteristics at index hospitalizations for liver transplantation, stratified by the presence of subsequent GVHD

	No GVHD (N=88,050)	GVHD (N=383)	*P*
Age (y)	57 (49–63)	58 (47–65)	0.338
Female (%)	35.4	43.7	0.282
Elixhauser comorbidity index	5 (4–7)	5 (4–7)	0.977
Median household income in patient’s zip code (%)			0.402
$1–$42,999	24.2	29.5	
$43,000–$53,999	26.2	29.1	
$54,000–$70,999	26.7	21.0	
$≥71,000	23.0	20.3	
Insurance (%)			0.192
Medicare	31.1	39.4	
Medicaid	17.2	18.2	
Private	50.5	38.6	
Self-pay	1.2	3.9	
Types of liver disease (%)			
Viral hepatitis	33.0	28.1	0.369
Alcohol-associated liver disease	29.0	19.0	0.023
Autoimmune hepatitis	4.4	4.2	0.918
Acute liver failure	9.1	6.0	0.142
Primary sclerosing cholangitis^a^	2.3	1.1	0.466
Primary liver cancer	27.2	25.7	0.753
Secondary diagnosis of COVID-19 on index admission (%)^b^	0.6	9.7	<0.001

a Only after September of 2015, because there was no specific code for primary sclerosing cholangitis in the ICD-9 era.

b COVID-19 as a secondary diagnosis during hospitalization of liver transplantation in 2020.

Abbreviations: GVHD, graft-versus-host disease; ICD-9, International Classification of Diseases 9th Revision.

### Diagnoses and procedures on GVHD hospitalizations

The frequency of ICD-9 and ICD-10 diagnoses, other than GVHD, during hospitalizations for GVHD, was ranked to create a comprehensive secondary diagnostic profile (Table 3 and Supplemental Table S3, http://links.lww.com/HC9/A536). Hematological conditions were especially prevalent, accounting for over 40% of hospitalizations, with specific diagnoses including other pancytopenia (284.19, 17.7%), unspecified neutropenia (288.00, 13.3%), and pancytopenia (28.41, 12.4%) in ICD-9 and other pancytopenia (D61.818, 48.5%) in ICD-10. Other frequently occurring diagnoses include acute kidney injury, electrolyte imbalances, sepsis or systemic inflammation, pleural effusion, and diarrhea. Essential hypertension was the most prevalent comorbidity, present in 36.7% of ICD-9 cases and 35.3% of ICD-10 cases.

**TABLE 3:** Frequency of principal diagnoses at index hospitalization for graft-versus-host disease following liver transplantation

	ICD-9			ICD-10		
Rank	ICD code	Description	Rate (%)	ICD code	Description	Rate (%)
1	99682	Complications of transplanted liver	66.9	T8649	Other complications of liver transplant	48.6
2	4019	Unspecified essential hypertension	36.7	D61818	Other pancytopenia	48.5
3	78959	Other ascites	24.3	I10	Essential (primary) hypertension	35.3
4	5849	Acute kidney failure, unspecified	23.1	Z944	Liver transplant status	34.2
5	5715	Cirrhosis of liver without mention of alcohol	21.1	E871	Hypoosmolality and hyponatremia	29.3
6	V441	Gastrostomy status	20.0	Y830	Surgical operation with transplant of whole organ	28.5
7	2761	Hypoosmolality	19.5	Z87891	Personal history of nicotine dependence	27.5
8	V4283	Pancreas transplant status	19.1	Z515	Palliative care	27.3
9	V427	Liver transplant status	19.1	E43	Unspecified severe protein-energy malnutrition	27.2
10	V4284	Organ or tissue replaced by transplant, intestines	19.1	R6521	Severe sepsis with septic shock	26.6
11	5119	Pleural effusion, not otherwise specified	19.0	Z66	Do not resuscitate	25.9
12	414	Other forms of chronic ischemic heart disease	18.7	J90	Pleural effusion, not elsewhere classified	25.2
13	28419	Other pancytopenia	17.7	R197	Diarrhea, unspecified	24.1
14	V1091	Personal history of malignant neuroendocrine tumor	17.4	N179	Acute renal failure, unspecified	23.8
15	99687	Complications of transplanted intestine	17.4	A419	Sepsis, unspecified	23.5
16	28800	Neutropenia, unspecified	13.3	E872	Acidosis	23.0
17	51881	Acute respiratory failure	13.1	N170	Acute renal failure with tubular necrosis	21.5
18	2841	Pancytopenia	12.4	Z7982	Long-term (current) use of aspirin	18.6
19	5723	Portal hypertension	12.4	T380X5A	Adverse effect of glucocorticoids and synthetic analogues, initial encounter	18.4
20	2752	Disorders of magnesium metabolism	12.1	Z7952	Long-term (current) use of systemic steroids	18.0

Abbreviations: ICD, International Classification of Diseases; ICD-9, International Classification of Diseases, 9th Revision; ICD-10, International Classification of Diseases, 10th Revision.

Among these patients, 62.3% underwent a biopsy during their first GVHD hospitalization. Of these, 44.7% received a skin biopsy, 36.5% underwent a liver biopsy, and 46.7% had a biopsy of the gastrointestinal tract. No bleeding events were documented for skin biopsies. In contrast, 7.5% of liver biopsies and 9.9% of gastrointestinal tract biopsies reported bleeding events though these differences were not statistically significant (*p* = 0.567). Regarding mortality, the rates were 49.0% for skin biopsies, 26.3% for liver biopsies, and 35.5% for gastrointestinal tract biopsies, with no statistically significant difference (*p* = 0.537).

### Risk factors of GVHD

Several factors were associated with an increased risk of GVHD after LT (Table 4). Univariate analysis showed that AALD was a significant negative predictor of GVHD [HR: 0.57 (95% CI: 0.35–0.93, *p* = 0.024)]. This association was also confirmed in the multivariate analysis, where the HR remained unchanged at 0.57 (95% CI: 0.36–0.91, *p* = 0.018). Furthermore, secondary diagnosis of COVID-19 on index admission was found to be a significant predictor of GVHD with a HR of 21.30 (95% CI: 2.80–161.91, *p* = 0.003) in the univariate analysis and 22.31 (95% CI: 2.81–177.33, *p* = 0.003) in the multivariate analysis. Only a small proportion of LT hospitalizations had a secondary diagnosis of COVID-19 (0.7%, significantly lower than the 5.8% prevalence of COVID-19 in general all-cause hospitalizations, *p* < 0.001). Other factors such as age, sex, the Elixhauser comorbidity index, and the occurrence of typical diseases leading to an indication for LT were not found to be significant predictors of GVHD.

**TABLE 4:** Predictors of GVHD after liver transplant hospitalization

	Univariate analysis	Multivariate analysis
Age (y)	0.98 (0.97–1.00), 0.104	
Female (%)	1.42 (0.76–2.67), 0.275	
Elixhauser comorbidity index	0.90 (0.71–1.14), 0.383	
Diabetes mellitus	1.15 (0.72–1.82), 0.567	
Types of liver disease (%)		
Viral hepatitis	0.78 (0.47–1.30), 0.342	
Alcohol-associated liver disease	**0.57** (**0.35**–**0.93), 0.024**	**0.57** (**0.35**–**0.92), 0.022**
Autoimmune hepatitis	0.96 (0.41–2.28), 0.931	
Acute liver failure	0.65 (0.36–1.17), 0.150	
Primary sclerosing cholangitis^a^	0.51 (0.07–3.71), 0.509	
Primary liver cancer	0.93 (0.56–1.53), 0.773	
Secondary diagnosis of COVID-19 on index admission (%)	**21.30** (**2.80**–**161.91), 0.003**	**23.16** (**3.07**–**177.79), 0.002**

Numbers listed: HR values shown with 95% CI in parenthesis and *p* value after comma.

a Only after September of 2015, because there was no specific code for primary sclerosing cholangitis in the ICD-9 era.

Abbreviations: GVHD, graft-versus-host disease; ICD-9, International Classification of Diseases 9th Revision.

### The role of liver disease as a risk factor for GVHD after HSCT

We further analyzed liver disease as a potential risk factor for GVHD following HSCT. Between 2016 and 2020, 95,666 HSCT procedures were identified, with 14,668 of these cases developing GVHD within the same calendar year, representing an incidence rate of 38.8% (95% CI: 37.9%–39.8%). Liver disease was prevalent in 22.8% of HSCT patients who developed GVHD and 14.9% of those who did not (*p* < 0.001). On adjusting for variables such as age, sex, and the Elixhauser comorbidity index, liver disease emerged as a significantly associated factor for a higher GVHD risk post-HSCT (adjusted HR: 1.77, 95% CI: 1.62–1.94, *p* < 0.001; see Supplemental Table S4, http://links.lww.com/HC9/A536 for additional data).

## DISCUSSION

GVHD is a grave, yet uncommon complication that arises following LT. The mortality rate associated with GVHD can be as high as 80%–100%.^2,6^ Our study revealed a calendar-year all-cause mortality rate of 41.7% after hospitalization for GVHD. Among the survivors, at the conclusion of the calendar year, 19.1% had received consultations for palliative care. Collectively, these findings suggest that over 60% of the patients experienced a rapid decline in their health, ultimately leading to death shortly after the onset of GVHD. A systematic review of the literature through 2015 identified only 156 adult patients who developed GVHD after LT.^3^ Our study represents the first nationwide investigation of GVHD after LT. The incidence of GVHD within the first year after LT in the United States was 1.0%. Despite yearly fluctuations, the overall trend of GVHD after LT remained stable from 2010 to 2020. During hospitalizations related to GVHD, abnormalities such as pancytopenia, leukocytopenia, acute kidney injury, electrolyte disturbances, sepsis, systemic inflammation, pleural effusions, and diarrhea are commonly observed. Billingham^18^ described the essential requirement for the development of GVHD, which includes the following: (1) the graft must contain immunologically competent cells; (2) the recipient must be recognized as foreign by the graft; and (3) the recipient must be unable to reject the graft before it mounts an effective immune response.^19^ Previous research has identified several risk factors for the development of GVHD after LT, including recipient-donor age discrepancy >20 years, human leukocyte antigen mismatch, younger donor age, recipient age >50 years, and glucose intolerance.^3,20^ Our results demonstrated that patients with AALD undergoing LT have a lower probability of developing GVHD post-transplant. In addition, we showed that a secondary diagnosis of COVID-19 on index admission was associated with an elevated risk of GVHD.

AALD was an indication for 32.1% of LTs.^14^ Previous studies have documented that AALD was the second most common indication for LT among patients who developed GVHD, accounting for 22.9% of cases.^3^ Similarly, our study found that patients with AALD had a decreased probability of GVHD after LT. There is no evidence, but several hypotheses might support this phenomenon. First, compared with AALD, the immune system plays a larger role in other etiologies leading to liver transplants, such as viral hepatitis, NASH, and autoimmune diseases; this might result in more host antigen-presenting cells activating the donor immune cells. Second, both ethanol and its metabolic byproduct, acetaldehyde, have direct toxic effects on liver cells in AALD. The hepatocytes release damage-associated molecular patterns, which in turn recruit immune cells and cause inflammation. This inflammation leads to fatty acid oxidation and steatosis, which further worsen liver damage. However, direct chemical damage subsides when alcohol consumption is stopped, which is typically done before LT. The period of alcohol abstinence is believed to have a positive impact on the recovery from hepatocellular injury by removing the triggers for inflammation, oxidative stress, and steatosis. In contrast, patients undergoing LT for other reasons may not have this advantage. The complete removal of the harmful substance causing liver injury may explain the relatively lower risk of GVHD in AALD patients receiving LT, although further investigation is needed to understand the underlying mechanism.^21^ Third, alcohol use is associated with an immunosuppressed state, which involves structural host defense mechanisms in the gastrointestinal and respiratory tract, as well as all of the main components of the innate and adaptive immune systems.^22^ The immunosuppressed state, associated with GVHD, usually can improve during the alcohol abstinence period before the LT too. Of note is the implementation of alcohol abstinence protocols at transplant centers. These abstinence programs typically include a mandatory 6-month period of sobriety before transplant evaluation, psychosocial assessments to determine the risk of relapse and appropriateness of transplant, and completion of a chemical dependence program as a prerequisite to transplant.^23^ This ensures that only patients who are most likely to persistently abstain from alcohol are eligible for LT. Early LT in AALD is a topic of great interest within the field of hepatology,^23–26^ and the incidence of GVHD in early recipients of LT in this population needs to be monitored further to ensure that the benefit of decreased GVHD incidence is preserved regardless of the duration of transplantation.

This study represents the first demonstration of a correlation between COVID-19 infection and increased risk of GVHD. The current pandemic has revealed a significant incidence of hepatic dysfunction in 14%–53% of patients with COVID-19, particularly among those with severe disease.^27^ Severe acute respiratory syndrome coronavirus 2 (SARS-CoV-2) uses angiotensin-converting enzyme 2 as a receptor to gain access to host cells.^28^ Angiotensin-converting enzyme 2 receptors’ expression is widely distributed in the gastrointestinal tract, vascular endothelium, and liver cholangiocytes.^27^ The development of post-LT GVHD involves a preexisting condition of immunocompromise and inflammation that enhances the function of host antigen-presenting cells by upregulating the expression of major histocompatibility complex classes I and II. After LT, the transfer of immunocompetent donor leukocytes results in the activation of passenger lymphocytes when they interact with host antigen-presenting cells that express upregulated human leukocyte antigen peptides. In the context of major histocompatibility complex mismatch following LT, activated lymphocytes undergo clonal expansion that is dependent on IL-2. This expansion process promotes the generation of memory cells and cytotoxic T cells.^29^ In the case of SARS-CoV-2 infection, the tissue damage and the subsequent viral-induced cytokine storm can establish a proinflammatory state characterized by increased levels of IL-1, IL-2, IL-6, and TNF-α.^30^ These elevated cytokine levels may contribute to the development of GVHD. Given the evolution of SARS-CoV-2 over time, it is of paramount importance to continuously evaluate the correlation between the presence of different viral strains and their potential to elicit a similar GVHD response. However, we did not observe a significant increase in the incidence of GVHD in 2020, likely because only a small proportion of patients hospitalized for LT admission had COVID-19 infection. This theory is supported by our finding that the prevalence of secondary diagnosis of COVID-19 on index admission was significantly lower than the prevalence of COVID-19 in general hospitalizations (0.7% vs. 5.8%, respectively; OR: 0.11, *p* < 0.001). This disparity is likely explained by the national practice pattern of avoiding LT, while recipient patients have an active COVID-19 infection.

We also explored the context of biopsy procedures in the management of GVHD. We found that a significant proportion of patients underwent a biopsy during their first GVHD hospitalization. Among these, skin biopsies were most frequent, followed by gastrointestinal tract and liver biopsies. This ordering might reflect clinicians’ choices based on perceived risk, as no bleeding events were reported for skin biopsies, while liver and gastrointestinal tract biopsies carried some bleeding risk. The mortality rates were similar across these types of biopsies, suggesting that the overall risk may not be significantly different.

Our study made an additional contribution by further dissecting the ICD-10 D89.81 subcodes to gain more insight into the nature of GVHD after LT. We found that 37.0% of the first episodes of GVHD after LT were acute, 6.6% were chronic, 1.2% were acute or chronic, and the remaining 55.2% were unspecified. Our data indicate that the majority of GVHD cases after LT were either acute or unspecified, most of which manifested within 3 months of transplantation. This finding underscores the critical need for close patient monitoring during the initial months following LT.

In addition to the factors discussed above, our supplementary analysis explored the role of liver disease as a potential risk factor in the development of GVHD in nonliver transplant patients, particularly those undergoing HSCT. Given the high incidence of GVHD observed in HSCT, we focused our examination on this subset and found a significant association between the presence of liver disease and higher GVHD development risk post-HSCT. This analysis, which utilized data from 2016 to 2020, enhances our understanding of GVHD’s nuances across various transplant contexts and underscores the need for concerted research efforts to mitigate the risk factors inherent in these clinical scenarios. These insights, derived from our own data, fill a vital gap in the available literature on this important topic.

The current study had some limitations that must be considered in its interpretation. The NRD comprises data from ~18 million hospital discharges annually across 28 states in the United States, and national estimates are generated using a weighted methodology. Therefore, the findings reported, including cases of liver LT, are national estimates and may exhibit minor deviations from registries that include unweighted cases such as the UNOS. Given that UNOS does not provide access to data on all GVHD cases, we contend that NRD is a valuable alternative for research in this field. Second, the use of ICD-9/10 codes as a means of identifying diseases, procedures, and comorbidities within the NRD database may result in diagnostic misclassification. Nonetheless, we employed high-validity ICD-9/10 procedural codes to identify LT, and our results demonstrated comparable yearly LT volume and fatal GVHD case numbers as reported by UNOS. Currently, no alternative methods exist to evaluate the incidence and trend of post-LT GVHD at the national level other than through administrative databases. Third, the lack of laboratory results and donor information in the NRD database restricts a more comprehensive analysis. This includes some specific variables such as recipient-donor age discrepancy, human leukocyte antigen mismatch, and younger donor age, which are known risk factors for the development of GVHD after LT. To compensate for this, we incorporated the Elixhauser comorbidity index to account for the complexity of chronic illnesses. Fourth, the NRD does not enable the tracking of patients past the end of each calendar year, so the incidence rate of GVHD in this analysis reflects only the first year after LT. During LT, an estimated 10^9^–10^10^ donor lymphoid cells are transplanted along with the liver graft.^31^ As a result, chimerism, the coexistence of donor and recipient cells, commonly develops within a span of 3–4 weeks after LT. Thus, GVHD predominantly occurs early after LT.^19^ This pathophysiologic fact justifies the use of the NRD, as the vast majority of GVHD after LT should occur well under the 1 calendar year period. Further research employing alternative methods is necessary to evaluate the incidence rate beyond the first year, which we suspect is lower based on previous studies. Fifth, the mortality rate of GVHD was likely underestimated in our study because of the occurrence of deaths outside hospitalization, which would remain undocumented in the NRD. To address this limitation, we reported the frequency of patients receiving palliative consultation as a surrogate marker for outpatient and hospice mortality. This approach provides a more comprehensive picture of mortality rates associated with GVHD than those documented in hospital records. Nonetheless, the use of palliative care consultation as a surrogate marker for postdischarge mortality should also be interpreted with caution. While palliative care consultation is often associated with advanced disease and mortality, the current practice of palliative care has expanded to managing symptoms and improving quality of life throughout the disease trajectory, regardless of the prognosis. Finally, it is important to note that due to the unavailability of data from years before the index admission, we were unable to exclude LT recipients who may have undergone multiple transplantations in the previous distinct calendar years. We anticipate that this issue is restricted to a minor subset of the patient population and thus will not significantly impact the validity of our analysis.

In conclusion, our study provides the first national estimate of the incidence and mortality rates of GVHD after LT in the United States. The incidence rate of GVHD within the first year after LT was 1.0%, with no significant trend from 2010 to 2020. Patients with AALD who underwent LT had a lower probability of developing GVHD post-transplantation. A secondary diagnosis of COVID-19 on index admission was associated with an elevated risk of GVHD. Hematological abnormalities related to GVHD were commonly observed during hospitalizations, with essential hypertension being the most prevalent comorbidity. The mortality rate associated with GVHD hospitalization was high, with over 40% of patients dying and an additional 20% receiving palliative care consult within 1 calendar year. Our study provides valuable insights into the incidence, risk factors, and outcomes of GVHD after LT in the United States.

## Supplementary Material

## References

[1] Rogulj IM Deeg J Lee SJ . Acute graft versus host disease after orthotopic liver transplantation. J Hematol Oncol. 2012;5:50. 22889203 10.1186/1756-8722-5-50 PMC3445845

[2] Chen XB Yang J Xu MQ Wen TF Yan LN . Unsuccessful treatment of four patients with acute graft-vs-host disease after liver transplantation. World J Gastroenterol. 2012;18:84–89. 22228975 10.3748/wjg.v18.i1.84 PMC3251810

[3] Murali AR Chandra S Stewart Z Blazar BR Farooq U Ince MN . Graft versus host disease after liver transplantation in adults: A case series, review of literature, and an approach to management. Transplantation. 2016;100:2661–2670. 27495762 10.1097/TP.0000000000001406 PMC5118135

[4] Taylor AL Gibbs P Sudhindran S Key T Goodman RS Morgan CH . Monitoring systemic donor lymphocyte macrochimerism to aid the diagnosis of graft-versus-host disease after liver transplantation. Transplantation. 2004;77:441–446. 14966423 10.1097/01.TP.0000103721.29729.FE

[5] Smith DM Agura E Netto G Collins R Levy M Goldstein R . Liver transplant-associated graft-versus-host disease. Transplantation. 2003;75:118–126. 12544883 10.1097/00007890-200301150-00022

[6] Perri R Assi M Talwalkar J Heimbach J Hogan W Moore SB . Graft vs. host disease after liver transplantation: A new approach is needed. Liver Transpl. 2007;13:1092–1099. 17663410 10.1002/lt.21203

[7] Chan EY Larson AM Gernsheimer TB Kowdley KV Carithers RL Reyes JD . Recipient and donor factors influence the incidence of graft-vs.-host disease in liver transplant patients. Liver Transpl. 2007;13:516–522. 17394149 10.1002/lt.21082

[8] Wu G Selvaggi G Nishida S Moon J Island E Ruiz P . Graft-versus-host disease after intestinal and multivisceral transplantation. Transplantation. 2011;91:219–224. 21076376 10.1097/TP.0b013e3181ff86ec

[9] Newell LF Dunlap J Gatter K Bagby GC Press RD Cook RJ . Graft-versus-host disease after liver transplantation is associated with bone marrow failure, hemophagocytosis, and DNMT3A mutations. Am J Transplant. 2021;21:3894–3906. 33961341 10.1111/ajt.16635

[10] Pahari H Nagai S Skorupski S Salgia R . Graft-versus-host disease of the central nervous system after liver transplantation: A rare complication. Am J Transplant. 2018;18:2591–2594. 29935052 10.1111/ajt.14981

[11] Knox KS Behnia M Smith LR Vance GH Busk M Cummings OW . Acute graft-versus-host disease of the lung after liver transplantation. Liver Transpl. 2002;8:968–971. 12360443 10.1053/jlts.2002.35552

[12] Strasser SI Shulman HM Flowers ME Reddy R Margolis DA Prumbaum M . Chronic graft-versus-host disease of the liver: Presentation as an acute hepatitis. Hepatology. 2000;32:1265–1271. 11093733 10.1053/jhep.2000.20067

[13] Kim GY Schmelkin LA Davis MDP el-Azhary RA Farrell AM Meves A . Dermatologic manifestations of solid organ transplantation-associated graft-versus-host disease: A systematic review. J Am Acad Dermatol. 2018;78:1097–1101.e1. 29288097 10.1016/j.jaad.2017.12.050 PMC6167008

[14] Kwong AJ Ebel NH Kim WR Lake JR Smith JM Schladt DP . OPTN/SRTR 2020 annual data report: Liver. Am J Transplant. 2022;22(suppl 2):204–309. 35266621 10.1111/ajt.16978

[15] Overview of the Nationwide Readmissions Database (NRD). Accessed January 29, 2023. https://www.hcup-us.ahrq.gov/nrdoverview.jsp

[16] DUA Training—Accessible Version. Healthcare cost and utilization project data use agreement course. Accessed January 2, 2023. https://www.hcup-us.ahrq.gov/DUA/dua_508/DUA508version.jsp

[17] Elixhauser A Steiner C Harris DR Coffey RM . Comorbidity measures for use with administrative data. Medical Care. 1998;36:8–27. 9431328 10.1097/00005650-199801000-00004

[18] Billingham RE . The biology of graft-versus-host reactions. Harvey Lect. 1996;62:21–78. 4875305

[19] Chaib E Silva FD Figueira ERR Lima FR Andraus W D'Albuquerque LAC . Graft-versus-host disease after liver transplantation. Clinics (Sao Paulo). 2011;66:1115–1118. 21808887 10.1590/S1807-59322011000600035 PMC3129945

[20] Kitajima T Henry M Ivanics T Yeddula S Collins K Rizzari M . Incidence and risk factors for fatal graft-versus-host disease after liver transplantation. Transplantation. 2021;105:2571–2578. 33449608 10.1097/TP.0000000000003607

[21] Dunn W Shah VH . Pathogenesis of alcoholic liver disease. Clin Liver Dis. 2016;20:445–456. 27373608 10.1016/j.cld.2016.02.004 PMC4933837

[22] Molina PE Happel KI Zhang P Kolls JK Nelson S . Focus on: Alcohol and the immune system. Alcohol Res Health. 2010;33:97–108. 23579940 PMC3887500

[23] Weinberg EM Dukewich M Jakhete N Stonesifer E Im GY Lucey MR . Early liver transplantation for severe alcohol-associated hepatitis and a history of prior liver decompensation. Am J Gastroenterol. 2022;117:1990–1998. 35853462 10.14309/ajg.0000000000001901 PMC10361649

[24] Mathurin P Moreno C Samuel D Dumortier J Salleron J Durand F . Early liver transplantation for severe alcoholic hepatitis. N Engl J Med. 2011;365:1790–1800. 22070476 10.1056/NEJMoa1105703

[25] Lee BP Mehta N Platt L Gurakar A Rice JP Lucey MR . Outcomes of early liver transplantation for patients with severe alcoholic hepatitis. Gastroenterology. 2018;155:422–430.e1. 29655837 10.1053/j.gastro.2018.04.009 PMC6460480

[26] Louvet A Labreuche J Moreno C Vanlemmens C Moirand R Féray C . Early liver transplantation for severe alcohol-related hepatitis not responding to medical treatment: A prospective controlled study. Lancet Gastroenterol Hepatol. 2022;7:416–425. 35202597 10.1016/S2468-1253(21)00430-1

[27] Jothimani D Venugopal R Abedin MF Kaliamoorthy I Rela M . COVID-19 and the liver. J Hepatol. 2020;73:1231–1240. 32553666 10.1016/j.jhep.2020.06.006 PMC7295524

[28] Li W Moore MJ Vasilieva N Sui J Wong SK Berne MA . Angiotensin-converting enzyme 2 is a functional receptor for the SARS coronavirus. Nature. 2003;426:450–454. 14647384 10.1038/nature02145 PMC7095016

[29] Wood A Eghtesad B Lindenmeyer CC . Graft-versus-host disease after liver transplantation. Clin Liver Dis (Hoboken). 2020;15:81–84. 32226622 10.1002/cld.884 PMC7098667

[30] Ye Q Wang B Mao J . The pathogenesis and treatment of the “Cytokine Storm” in COVID-19. J Infect. 2020;80:607–613. 32283152 10.1016/j.jinf.2020.03.037 PMC7194613

[31] Ferrara JLM . Pathogenesis of acute graft-versus-host disease: Cytokines and cellular effectors. J Hematother Stem Cell Res. 2000;9:299–306. 10894351 10.1089/15258160050079407

