# Supplementary material for: Incidence and risk factors of graft-versus-host disease after liver transplantation: A national study 2010–2020
Source: Hepatol Commun. 2023 Sep 27;7(10):e0271. doi: 10.1097/HC9.0000000000000271 (PMC10531268; doi:10.1097/HC9.0000000000000271)
Supplement: Supplementary file 1 [file hc9-7-e0271-s001.docx]

| **Supplement Table 1**. ICD Codes for Diagnosis and Procedures | | |
| --- | --- | --- |
| Condition | ICD-9 | ICD-10 |
| Liver transplantation | 50.5* | 0FY00Z0 |
| Graft-versus-host disease | 279.5* | D89.81* |
| Viral hepatitis | 070.* | B15.*, B16.*, B17.*, B18.*, B19.* |
| Alcohol-related liver disease | 571.0*, 571.1*, 571.2*, 571.3* | K70.* |
| Auto immune hepatitis | 571.42 | K75.4* |
| Hepatocellular carcinoma | 155.* | C22.* |
| Acute liver failure | 570* | K72.0* |
| Primary sclerosing cholangitis | - | K83.01* |
| COVID-19 | - | U07.1* |
| Palliative care | V667* | Z51.5* |
| Hematopoietic stem cell transplantation | - | 30243A*, 30243C*, 30243G*, 30243U*, 30243X*, 30243Y*, 30233A*, 30233C*, 30233G*, 30233U*, 30233X*, 30233Y* |
| Liver disease | - | B15*, B16*, B17*, B18*, B19*, K7* |
| Skin biopsy | - | 0HB0*, 0HB1*, 0HB2*, 0HB3*, 0HB4*, 0HB5*, 0HB6*, 0HB7*, 0HB8*, 0HB9*, 0HBA*, 0HBB*, 0HBC*, 0HBD*, 0HBE*, 0HBF*, 0HBG*, 0HBH*, 0HBJ*, 0HBK*, 0HBL*, 0HBM*, 0HBN* |
| Liver biopsy | - | 0FB0*, 0FB1*, 0FB2* |
| Biopsy of gastrointestinal tract | - | 0DB1*, 0DB2*, 0DB3*, 0DB4*, 0DB5*, 0DB6*, 0DB7*, 0DB8*, 0DB9*, 0DBA*, 0DBB*, 0DBC*, 0DBE*, 0DBF*, 0DBG*, 0DBH*, 0DBJ*, 0DBK*, 0DBL*, 0DBM*, 0DBN*, 0DBP*, 0DBQ*, 0DBR* |
| "*" is used as a wildcard character in ICD codes, indicating that any additional digits can be used. There was no specific code for COVID-19 and primary sclerosing cholangitis in ICD-9 era. Abbreviations: ICD, International Classification of Diseases; COVID-19, coronavirus disease 2019 | | |

| **Supplement Table 2**. Risk of graft versus host disease after liver transplantation in different years | | | | |
| --- | --- | --- | --- | --- |
| Year | Hazard ratio | 95% confidence interval | | P |
| 2010 | Reference | Reference | Reference | Reference |
| 2011 | 0.52 | 0.19 | 1.39 | 0.193 |
| 2012 | 0.25 | 0.08 | 0.77 | 0.016 |
| 2013 | 0.31 | 0.09 | 1.06 | 0.063 |
| 2014 | 0.26 | 0.09 | 0.76 | 0.013 |
| 2015 | 0.23 | 0.07 | 0.78 | 0.019 |
| 2016 | 0.44 | 0.16 | 1.25 | 0.125 |
| 2017 | 0.35 | 0.14 | 0.91 | 0.031 |
| 2018 | 0.47 | 0.17 | 1.29 | 0.143 |
| 2019 | 0.33 | 0.12 | 0.90 | 0.030 |
| 2020 | 0.36 | 0.14 | 0.90 | 0.029 |

| **Supplement Table 3**. All diagnosis on the first graft-versus-host admission after index hospitalization of liver transplantation.* | | | | | | |
| --- | --- | --- | --- | --- | --- | --- |
|  | ICD-9 | | | ICD-10 | | |
| Rank | ICD code | Count | Rate | ICD code | Count | Rate |
| 1 | 99682 | 93 | 66.9% | D89813 | 90 | 64.0% |
| 2 | 27950 | 88 | 63.4% | T8649 | 68 | 48.6% |
| 3 | 4019 | 51 | 36.7% | D61818 | 68 | 48.5% |
| 4 | 78959 | 34 | 24.3% | I10 | 50 | 35.3% |
| 5 | 5849 | 32 | 23.1% | Z944 | 48 | 34.2% |
| 6 | 5715 | 29 | 21.1% | D89810 | 43 | 30.9% |
| 7 | V441 | 28 | 20.0% | E871 | 41 | 29.3% |
| 8 | 2761 | 27 | 19.5% | Y830 | 40 | 28.5% |
| 9 | V427 | 27 | 19.1% | Z87891 | 39 | 27.5% |
| 10 | V4283 | 27 | 19.1% | Z515 | 38 | 27.3% |
| 11 | V4284 | 27 | 19.1% | E43 | 38 | 27.2% |
| 12 | 5119 | 26 | 19.0% | R6521 | 37 | 26.6% |
| 13 | 414 | 26 | 18.7% | Z66 | 36 | 25.9% |
| 14 | 28419 | 25 | 17.7% | J90 | 35 | 25.2% |
| 15 | 27952 | 24 | 17.4% | R197 | 34 | 24.1% |
| 16 | 99687 | 24 | 17.4% | N179 | 33 | 23.8% |
| 17 | V1091 | 24 | 17.4% | A419 | 33 | 23.5% |
| 18 | 27951 | 23 | 16.7% | E872 | 32 | 23.0% |
| 19 | 28800 | 18 | 13.3% | N170 | 30 | 21.5% |
| 20 | 51881 | 18 | 13.1% | Z7982 | 26 | 18.6% |
| 21 | 2841 | 17 | 12.4% | T380X5A | 26 | 18.4% |
| 22 | 5723 | 17 | 12.4% | Z7952 | 25 | 18.0% |
| 23 | 2752 | 17 | 12.1% | R21 | 23 | 16.5% |
| 24 | 2639 | 16 | 11.8% | E8342 | 23 | 16.3% |
| 25 | 45621 | 15 | 11.1% | D709 | 22 | 15.8% |
| 26 | 99592 | 15 | 11.1% | J9601 | 22 | 15.8% |
| 27 | 2762 | 15 | 10.8% | T8641 | 22 | 15.6% |
| 28 | 7907 | 15 | 10.6% | Z8505 | 22 | 15.5% |
| 29 | 5762 | 14 | 10.3% | R5081 | 21 | 15.0% |
| 30 | 42731 | 14 | 10.0% | D638 | 20 | 14.6% |
| 31 | 2767 | 14 | 9.9% | Z79899 | 20 | 14.5% |
| 32 | 5990 | 14 | 9.8% | B370 | 20 | 14.3% |
| 33 | 2869 | 14 | 9.8% | Z681 | 20 | 14.2% |
| 34 | 25000 | 14 | 9.7% | K766 | 20 | 14.1% |
| 35 | 2875 | 13 | 9.6% | R188 | 19 | 13.8% |
| 36 | 389 | 13 | 9.1% | Z794 | 19 | 13.5% |
| 37 | 2859 | 12 | 8.9% | Z880 | 19 | 13.4% |
| 38 | 27651 | 11 | 8.2% | E870 | 18 | 12.9% |
| 39 | 2768 | 11 | 7.9% | J9811 | 17 | 12.2% |
| 40 | 2851 | 11 | 7.8% | I959 | 17 | 12.2% |
| 41 | 7071 | 11 | 7.6% | D649 | 17 | 12.0% |
| 42 | 99685 | 11 | 7.6% | E119 | 17 | 11.9% |
| 43 | 28749 | 11 | 7.6% | E8339 | 17 | 11.9% |
| 44 | 72252 | 11 | 7.6% | I2510 | 17 | 11.8% |
| 45 | 20511 | 11 | 7.6% | J9600 | 16 | 11.7% |
| 46 |  |  |  | R509 | 16 | 11.4% |
| 47 |  |  |  | E669 | 16 | 11.2% |
| 48 |  |  |  | Z86718 | 16 | 11.1% |
| 49 |  |  |  | E875 | 15 | 10.8% |
| 50 |  |  |  | E876 | 15 | 10.8% |
| 51 |  |  |  | R739 | 15 | 10.7% |
| 52 |  |  |  | A0472 | 15 | 10.7% |
| 53 |  |  |  | D62 | 15 | 10.4% |
| 54 |  |  |  | E874 | 15 | 10.4% |
| 55 |  |  |  | K529 | 14 | 10.1% |
| 56 |  |  |  | E8770 | 14 | 9.9% |
| 57 |  |  |  | D696 | 14 | 9.8% |
| 58 |  |  |  | E8351 | 13 | 9.6% |
| 59 |  |  |  | T451X5A | 13 | 9.4% |
| 60 |  |  |  | L308 | 13 | 9.4% |
| 61 |  |  |  | E1165 | 13 | 9.3% |
| 62 |  |  |  | K219 | 13 | 9.2% |
| 63 |  |  |  | Z888 | 13 | 9.2% |
| 64 |  |  |  | E785 | 13 | 9.1% |
| 65 |  |  |  | I480 | 13 | 9.1% |
| 66 |  |  |  | J156 | 13 | 9.1% |
| 67 |  |  |  | D689 | 13 | 9.1% |
| 68 |  |  |  | J189 | 13 | 9.0% |
| 69 |  |  |  | E1122 | 12 | 8.9% |
| 70 |  |  |  | Z9081 | 12 | 8.8% |
| 71 |  |  |  | E860 | 12 | 8.6% |
| 72 |  |  |  | K921 | 12 | 8.6% |
| 73 |  |  |  | B3781 | 12 | 8.5% |
| 74 |  |  |  | I129 | 12 | 8.4% |
| 75 |  |  |  | K567 | 12 | 8.3% |
| 76 |  |  |  | E46 | 12 | 8.3% |
| 77 |  |  |  | K3189 | 11 | 8.1% |
| 78 |  |  |  | Y848 | 11 | 8.0% |
| 79 |  |  |  | Z9483 | 11 | 7.9% |
| 80 |  |  |  | G9341 | 11 | 7.9% |
| 81 |  |  |  | E440 | 11 | 7.9% |
| 82 |  |  |  | G8929 | 11 | 7.8% |
| 83 |  |  |  | Z7901 | 11 | 7.7% |
| 84 |  |  |  | L309 | 11 | 7.6% |
| 85 |  |  |  | B1920 | 11 | 7.5% |
| *Diagnosis with ten or less cases are not reported in compliance to Healthcare Cost and Utilization Project Data Use Agreement. https://www.hcup-us.ahrq.gov/DUA/dua_508/DUA508version.jsp Abbreviations: ICD, International Classification of Diseases. | | | | | | |

| **Supplement Table 4**. Predictors of Graft-versus-Host Disease After Hematopoietic Stem Cell Transplant Hospitalization. | | |
| --- | --- | --- |
|  | Univariate analysis | Multivariate analysis |
| Liver disease | 1.75 (1.60-1.92), <0.001 | 1.77 (1.62-1.94), <0.001 |
| Age (years) | 0.99 (0.98-0.99), <0.001 | 0.99 (0.99-0.99), <0.001 |
| Female (%) | 1.04 (0.98-1.09), 0.191 | 1.05 (0.99-1.11), 0.098 |
| Elixhauser comorbidity index | 0.93 (0.91-0.95), <0.001 | 0.95 (0.93-0.97), <0.001 |
| Numbers listed: Hazard ratio values shown with 95% confidence interval in parenthesis and P-value after comma. *Only after September of 2015, because there was no specific code for primary sclerosing cholangitis in ICD-9 era. Abbreviations: COVID-19, coronavirus disease 2019; GVHD, graft-versus-host disease. | | |
